# First-Principles Investigation of Ag-Doped Gold Nanoclusters

**DOI:** 10.3390/ijms12052972

**Published:** 2011-05-09

**Authors:** Xiao-Dong Zhang, Mei-Li Guo, Di Wu, Pei-Xun Liu, Yuan-Ming Sun, Liang-An Zhang, Yi She, Qing-Fen Liu, Fei-Yue Fan

**Affiliations:** 1 Tianjin Key Laboratory of Molecular Nuclear Medicine, Institute of Radiation Medicine, Chinese Academy of Medical Sciences and Peking Union Medical College, Tianjin 300192, China; E-Mails: wudi521wan@163.com (D.W.); pharm8888@yahoo.com.cn (P.-X.L.); yuanmings1962@163.com (Y.-M.S.); zhangla43@yahoo.com.cn (L.-A.Z.); yi_she2005@yahoo.com.cn (Y.S.); qingfenliu@yahoo.com.cn (Q.-F.L.); 2 Department of Physics, Tianjin Institute of Urban Construction, Tianjin 300384, China; E-Mail: guomeili@tjuci.edu.cn

**Keywords:** first-principles, gold clusters, electronic structure, optical properties

## Abstract

Gold nanoclusters have the tunable optical absorption property, and are promising for cancer cell imaging, photothermal therapy and radiotherapy. First-principle is a very powerful tool for design of novel materials. In the present work, structural properties, band gap engineering and tunable optical properties of Ag-doped gold clusters have been calculated using density functional theory. The electronic structure of a stable Au_20_ cluster can be modulated by incorporating Ag, and the HOMO–LUMO gap of Au_20−_*_n_*Ag*_n_* clusters is modulated due to the incorporation of Ag electronic states in the HOMO and LUMO. Furthermore, the results of the imaginary part of the dielectric function indicate that the optical transition of gold clusters is concentration-dependent and the optical transition between HOMO and LUMO shifts to the low energy range as the Ag atom increases. These calculated results are helpful for the design of gold cluster-based biomaterials, and will be of interest in the fields of radiation medicine, biophysics and nanoscience.

## 1. Introduction

Gold nanostructures have attracted considerable attention owing to their unique electronic and optical properties, as well as their great potential for medical applications [1–3]. Today, gold nanostructures are also promising for their possible applications in photothermal therapy, radiotherapy and cancer cell imaging because of their good biocompatibility [4–6]. However, a disadvantage of photothermal therapy is that the optical absorption of gold nanoparticles, well known surface plasmon resonance (SPR), is always located in the visible light region, which limits the wider application of photothermal therapy *in vivo*. To utilize the high optical transmission of biotissue, it is necessary to enhance the near-infrared (NIR) optical absorption. In recent years, several groups have developed the gold nanorod, nanocage and core/shell structure, which are recognized as the promising materials for photothermal therapy [1,7,8]. However, the increasing size would obviously reduce the permeability of tumor tissue, which is disadvantageous to photothermal therapy.

Small gold clusters of typically 1–2 nm are proposed as alternative materials. Sun *et al.* have shown that the Au–SiO_2_ clusters can induce the unusual optical transition, and the optical absorption can be modulated to the NIR. This is also confirmed by the recent photothermal therapy and drug delivery experiment [9]. However, the gold clusters are structurally unstable and have ambiguous electronic structure. The procedure of designing gold clusters with NIR optical absorption is influenced obviously, because of the long time controversy, by the structure of gold clusters. Recently, the obvious progressing has been achieved by density functional theory (DFT) theory calculations and optical spectra experiments [10,11]. The common view is that gold clusters prefer the two dimension structure in the range of Au_4_–Au_13_, while the Au_14_–Au_20_ show the cage-like three dimension structure [12]. The more complex cage-like Au_32_–Au_38_ clusters have been predicted, although the experimental result may always be contradicted. The optical absorption of Au_2_–Au_13_, Au_19_, and Au_20_ have been calculated by time-dependent density functional theory (TDDFT), while the optical transition of Au_32_ is also focused due to its more stable structure. Notably, the tetrahedral Au_20_ cluster shows a band gap of ∼1.818 eV, and shows slight NIR absorption [13,14].

Doping Au clusters by other metals provides an available route to modulate electronic and optical properties [15]. It has been demonstrated that metal atoms, such as Zn and Cu, used to dope Au clusters can modify both their structural stability and optical properties due to the delocalizing *s* and *d* electronic configurations [16]. Bonacic-Koutecky *et al*. studied the structure and electronic properties of bimetallic Ag*_M_*Au*_N_* (3 < (M + N) < 5) clusters with DFT calculations [17]. For these clusters, the charge transferred from Ag to Au plays a dominant role in the structure of the bimetallic clusters. The Au–Ag bond is preferred to the Au–Au or Ag–Ag bond [18]. In addition, the metal-doped Au clusters may modulate the HOMO−LUMO gap [19,20]. Thus, we are interested in whether AuAg cluster has tunable optical absorption properties.

Here, we studied electronic structure and optical properties of Ag-doped Au_20_ clusters. The paper is organized as follows. Section 2 presents and discusses the results of our calculations. First, we investigated the structural properties by analyzing the binding energy. Then we calculated the electronic structures, because the optical properties depend on both the interband and intraband transitions, which are determined by electronic states. Finally, we analyzed the optical transition in different configurations. Section 3 describes the basic ingredients and details of computational methods we applied. Section 4 concludes and summarizes our findings.

## 2. Results and Discussion

### 2.1. Structural Properties of Ag-Doped Gold Clusters

**Figure 1.**
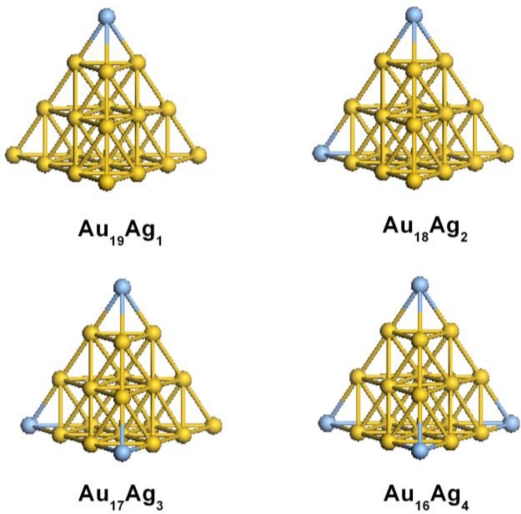
Calculated ground state geometries of Au_20_*_−n_*Ag*_n_*.

Figure 1 gives the calculated ground state geometries of Au_20_*_−n_*Ag*_n_*. The stability of gold nanostructure is very important for its electronic and optical properties. Thus, the binding energy per atom *E_b_*(*n*) of gold clusters is calculated to evaluate the stability of gold clusters, which are defined by the following formula:

(1) $$E_{b}(n)=\frac{E(\text{Ag})+nE(\text{Au})-nE({\text{Au}}_{n}\text{Ag})}{n+1}$$

where *E*(Ag), *E*(Au), and *E*(Au*_n_*Ag) represent the total energies of the most stable Ag, Au, and AuAg clusters, respectively. It is worth pointing out that all of the clusters are found to prefer the lowest spin state. We find that the binding energy of Au_20_ is 2.40 eV, which is very close to the previous investigation of gold clusters [21]. It has been demonstrated that the binding energy increases with an increase in the size of gold clusters [11]. Increasing binding energy means increasing stability due to enhanced core electron configurations. After Ag doping, the binding energy of Au_19_Ag_1_, Au_18_Ag_2_, Au_17_Ag_3_, and Au_16_Ag_4_ are 2.68, 2.68, 2.68, and 2.69 eV, respectively. The increasing doping atom induces a tiny effect on the binding energy. It is worth noting that the binding energy of AuAg alloy is higher than the Au_20_. It shows that Ag atom incorporation can enhance the structural stability. Indeed, the Au–Ag bond is stronger than the Au–Au bond and gives an extra σ-bonding interaction by the overlap between the vacant Ag 4*p* and valence Au 6*s* (5*d*) orbital, which is very similar to the previous Ag-doped gold clusters [20].

### 2.2. Electronic Structure of Ag-Doped Au_20_ Clusters

**Figure 2.**
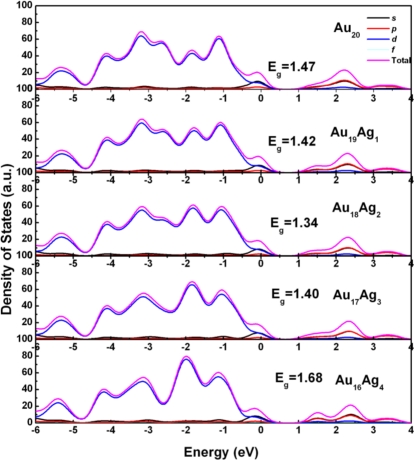
The partial DOS of (a) Au_20_*_−n_*Ag*_n_* clusters.

Figure 2 shows the density of states (DOS) to reveal the electronic structure of Au_20_*_−n_*Ag*_n_* clusters. The Au_20_ cluster shows the large HOMO-LUMO gap, which is in good agreement with the other computational results [10,21]. The exact band gap of Au_20_ is 1.47 eV, which is less than the experimental data of 1.78 eV (or 1.818 eV) due to the underestimation of electronic states by DFT [22]. Meanwhile, the Au *d* states are dominated in HOMO, and are located in the range of −6 and 0 eV. HOMO consists of Au *s* and *d* states; the Au *d* states are dominant. It is clearly seen that when *n* changes from 1 to 4, the DOS also changes. In general, the band gap of Au_20_*_−n_*Ag*_n_* clusters is less than that of the pure Au_20_ cluster expect for Au_16_Ag_4_. It confirms that the Ag incorporation into Au_20_ can induce the obvious effect on gap, which is consistent with the previous results [19,23,24]. The exact band gaps of Au_20_*_−n_*Ag*_n_* are 1.42, 1.34, 1.40, and 1.68 eV, which are corresponding to the different *n* values from 1 to 4. The variation of gap can be understood by electronic states. It can be seen that the LUMO of Au_19_Ag_1_, Au_18_Ag_2_, and Au_17_Ag_3_ is shifted to the low energy range compared with Au_20_, which can be clearly seen in Figure 2. This shift can lead to the obvious variation of electronic properties. As it is incorporated more and more, the *p* states in LUMO become stronger, which leads to the LUMO shift and band gap narrowing. However, the LUMO of Au_16_Ag_4_ shift to high energy range, and thus induce the increase of the gap, which should be related to improving structural stability and enclosing electronic configurations [25]. Meanwhile, the *p* and *s* states are obviously enhanced in the LUMO, and these electronic states can have effects on optical properties.

### 2.3. Tunable Optical Properties of Gold Clusters

**Figure 3.**
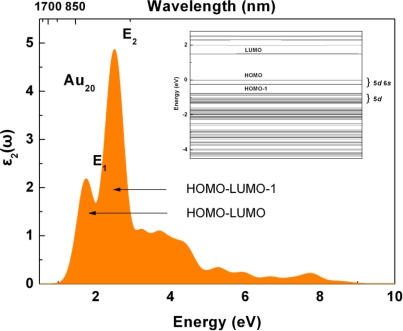
The imaginary part of dielectric function *ɛ*_2_(*ω*) of Au_20_ clusters.

For investigating the optical transition of Au_20_*_−n_*Ag*_n_* clusters, it is necessary to investigate the imaginary part of the dielectric function, because it is very important to the optical properties of any materials. Regarding Au_20_ in Figure.3, there are two main peaks in *ɛ*_2_(*ω*), at 1.79 and 2.51 eV, respectively (namely *E*_1_, *E*_2_), which are very close to the previous results of 1.86 and 2.78 [21]. It can be seen in the inset of Figure 3 that direct transition can be caused between HOMO and LUMO. Therefore, it can be expected that *E*_1_ (1.79 eV) should mainly be caused by optical transitions between Au 6*s* 55% (and Au *d*, 45%*)* states in HOMO and Au 6*s* states in LUMO, which are close to the other first-principles evaluation (1.86 eV) [21]. Moreover, it is not far from the HOMO-LUMO gap of 1.48 eV. In the DOS of Au_20_, the Au *d* states produce two peaks, 1.41 and 1.97 eV, which can induce some electronic states in the energy level range of −4∼0 eV. Thus, the optical transitions of *E*_2_ can be due to the optical transitions between HOMO-1 consist of Au *d* states and LUMO consist of Au 6*s* (and Au *p*) states.

**Figure 4.**
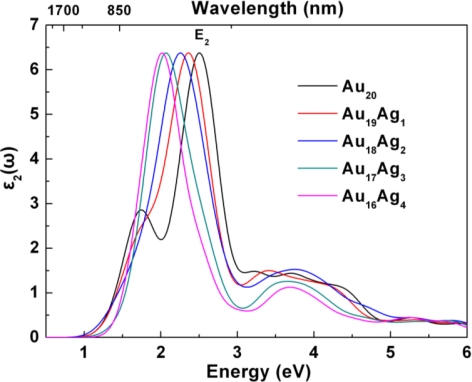
The tunable imaginary part of dielectric function *ɛ*_2_(*ω*) of Au_20_*_−n_*Ag*_n_* clusters.

Figure 4 shows the imaginary part of dielectric function *ɛ*_2_(*ω*) of Au_20_*_−n_*Ag*_n_* clusters. Ag incorporation induces some obvious variations of optical transitions. Firstly, *E*_1_ has gradually disappeared, which can be related to the red-shift of *E*_2_ and further inhibition of the intrinsic optical transition of *E*_1_. Secondly, *E*_2_ shows the tunable optical properties with the increasing Ag incorporation. The *E*_2_ of Au_20_, Au_19_Ag_1_, Au_18_Ag_2_, Au_17_Ag_3_, and Au_16_Ag_4_ is 2.51, 2.35, 2.25, 2.07, and 2.01 eV, respectively. To understand these optical phenomena in detail, it is necessary to analyze the optical transition by electronic states.

**Figure 5.**
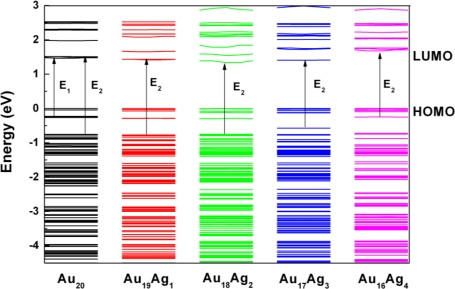
The outline of optical transition of Au_20_*_−n_*Ag*_n_* clusters calculated by energy level.

Figure 5 shows the transition energy level of Au_20_*_−n_*Ag*_n_* from band structure calculations. The optical transition between HOMO-LUMO has been affected by Ag incorporation. The variation in energy level can be described in two stages. In the first stage of Au_19_Ag_1_ and Au_18_Ag_2_, Ag electronic states contribute to both HOMO and LUMO. Furthermore, LUMO has slightly shifted to the low energy range, which induces the decrease of transition level and can be responsible for the red-shifts of E_2_. In the second stage of Au_17_Ag_3_ and Au_16_Ag_4_, the increasing Ag atom induces more dispersive in *d* states, and LUMO has shifted to the high energy range, which may cause gap variation. It has been supposed that the gap widening of Au_16_Ag_4_ is important evidence for the enhancement of binding energy and structural stability [25]. The heavy doping can have obvious effects on electronic states, and further investigation is still interesting. More valuable information is still necessary to probe by optical absorption.

**Figure 6.**
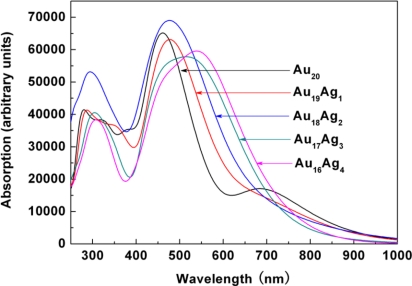
Optical absorption of Au_20_*_−n_*Ag*_n_* clusters.

Figure 6 presents the optical absorption of Au_20_*_−n_*Ag*_n_* clusters. The dominant absorption around 450–550 nm can be observed in all Au_20_*_−n_*Ag*_n_* clusters. For Au_20_, the absorption band of 450 nm could be due to the optical transition of *E*_2_, while the 707 nm absorption band is related to the intrinsic optical transition of *E*_1_. The Ag incorporation induces the red-shift of absorption band (*E*_2_) from 478 nm (Au_19_Ag_1_) to 543 nm (Au_16_Ag_4_). We need to consider two possible effects on the optical absorption of these Au_20_*_−n_*Ag*_n_* clusters. On the one hand, the HOMO-LUMO gap can be underestimated by the DFT. According to the available optical data of Au_20_ from the experiment, the gap of tetrahedral Au_20_ is about 1.7–1.8 eV, which is larger 0.23–0.33 eV than the calculated gap of 1.47 eV. Thus, the actual gap of Au_20_*_−n_*Ag*_n_* is wider than that of present calculated results. On the other hand, the structure stability of Au_20_*_−n_*Ag*_n_* should also be taken into account. In the geometry calculations, the binding energy of Au_16_Ag_4_ is higher than that of the pure Au_20_ cluster and the other AuAg clusters, which indicates the doping feasibility. Therefore, more Ag atom incorporation may be promising for further fabrication, NIR absorption and related applications. Zorriasatein *et al.* have proposed that Cu incorporation into Au clusters can modulate the band gap effectively, and the results showed that the introduction of Cu enhanced the binding energy per atom compared to Au clusters [25]. AuAg clusters also showed good structural stability and optical properties [23,26,27]. Thus, it can be expected that Ag incorporation is an effective strategy for modulating optical properties of Au clusters. Our results also clearly show that Ag incorporation can modify structural stability and modulate the optical properties of Au_20_ clusters. These methods also have potential applications in understanding the optical properties of metal nanoclusters and designing materials for photothermal therapy.

## 3. Computational Section

The calculations are based on density functional theory (DFT) using a plane-wave pseudopotential method [28]. We use the generalized gradient approximation (GGA) in the scheme of Perdew-Burke-Ernzerhof (PBE) to describe the exchange-correlation functional [29]. Norm-conserving pseudopotential is used to describe the electron-ion interaction. In this code, the plane wave functions of valence electrons are expanded in a plane wave basis set, and the use of norm-conserving pseudopotential allows a plane wave energy cutoff. Only plane waves with smaller kinetic energies are used in the expansion. Reciprocal-space integration over the Brillouin zone is approximated through careful sampling at a finite number of k-points using a Monkhorst-Pack mesh [30]. In the present system, full electron calculation for the Au_20_*_−n_*Ag*_n_* clusters is computationally rather expensive, so it is better to introduce effective core potentials for the Au and Ag atoms to describe the inner-core electrons. Under this approximation, the 5*d*^10^6*s*^1^ outermost valence electrons of the Au atom and 3*d*^10^4*s*^2^ outermost valence electrons of the Ag atom are described. It is well known that the interaction of a photon with the electrons in the system can be described in terms of time-dependent perturbations of the ground-state electronic states. Optical transitions between occupied and unoccupied states are caused by the electric field of the photon. The spectra from the excited states can be described as a joint density of states between the valence and conduction band. The momentum matrix elements, which are used to calculate the *ɛ*_2_(*ω*), are calculated between occupied and unoccupied states which are given by the eigen vectors obtained as solution of the corresponding Schrödinger equation. Evaluating these matrix elements, one uses the corresponding eigen functions of each of the occupied and unoccupied states [22,28,31]:

(2) $${ɛ}_{2}(\hbar \omega )=\frac{2e^{2}\pi }{\Omega {ɛ}_{0}}\sum_{k,v,c}{\left|\psi_{k}^{c}|u\cdot r|\psi_{k}^{v}〉\right|}^{2}\delta (E_{k}^{c}-E_{k}^{v}-\hbar \omega )$$

where Ω is the volume of the elementary cell, *v* and *c* represent the valence and conduction bands, respectively, *k* represents the *k* point, *ω* is the frequency of the incident light, and *u* is the vector defining the polarization of the electric field of the incident light, which is averaged over all spatial directions in the polycrystalline case. The real part of dielectric function *ɛ*_1_(*ω*) can be evaluated from the imaginary part *ɛ*_2_(*ω*) by the famous Kramer-Kronig relationship.

The calculations are performed at 20 × 20 Å supercell, which contains 20 neutral Au atoms. These models of gold clusters refer to the previous work, which has shown that the best structural stability and average distance between Au-Au bonding is about 2.73 Å [21]. The substitutional method has been taken into account in this paper, and Ag atoms are used to substitute Au atoms in vertex site. In this way, the Au_20_*_−n_*Ag*_n_* clusters are built. In order to weaken the interaction with Ag–Ag, the separation distance should be as far as possible. We choose the energy cutoff to be 720 eV, and the Brillouin-zone sampling mesh parameters for the k-point set are 2 × 2 × 2 for Au_20_*_−n_*Ag*_n_* clusters. The charge densities are converged to 2 × 10^−6^ eV/atom in the self-consistent calculation. And then, the Au_20−_*_n_*Ag*_n_* clusters are optimized with lattice constants and the positions of substitutional atoms. In the optimization process, the energy change, maximum force, maximum stress and maximum displacement tolerances are set as 2 × 10^−5^ eV/atom, 0.05 eV/Å, 0.1 GPa, and 0.002 Å, respectively.

## 4. Conclusions

In summary, a first-principles study has been performed to evaluate the electronic and optical properties of Au_20_*_−n_*Ag*_n_* clusters in different configurations. The Au_20_*_−n_*Ag*_n_* clusters show better binding energy and structural stability than the Au_20_ cluster. The increasing Ag concentration can induce the HOMO-LUMO gap variation. Subsequently, the optical transition between HOMO-LUMO has shifted to the low energy range with the increasing Ag concentration. Tunable optical transition has been observed, and was shown to decrease from 2.51 to 2.01 eV with the increase of Ag atoms. Our results clearly show that Ag incorporation can modulate the optical properties of Au_20_ clusters.
